# Skeletal Muscle Quality is Associated with Worse Survival After Pancreatoduodenectomy for Periampullary, Nonpancreatic Cancer

**DOI:** 10.1245/s10434-016-5495-6

**Published:** 2016-09-08

**Authors:** L. B. Van Rijssen, N. C. M. van Huijgevoort, R. J. S. Coelen, J. A. Tol, E. B. Haverkort, C. Y. Nio, O. R. Busch, M. G. Besselink

**Affiliations:** 1 Department of Surgery, Academic Medical Center, Amsterdam, The Netherlands; 2 Department of Nutrition and Dietetics, Academic Medical Center, Amsterdam, The Netherlands; 3 Department of Radiology, Academic Medical Center, Amsterdam, The Netherlands

## Abstract

**Background:**

Body composition measures may predict outcomes of cancer surgery. Whereas low muscle mass shown on preoperative computed tomography (CT) scans has been associated with worse outcomes after surgery for pancreatic cancer, less consideration has been given to low muscle attenuation, reflecting poor muscle quality. Studies relating muscle mass and muscle attenuation with outcomes for patients with periampullary, nonpancreatic cancer are lacking.

**Methods:**

Skeletal muscle mass and attenuation were assessed in 166 consecutive patients undergoing pancreatoduodenectomy (PD) for periampullary, nonpancreatic cancer at a single center between 2000 and 2012. The skeletal muscle index (SMI) was calculated from cross-sectional muscle area on preoperative CT imaging at the third lumbar vertebra level (L3) and normalized for height. The skeletal muscle attenuation index (MAI) was calculated by measuring the average Hounsfield units of the total muscle area at the L3 level. Overall survival (OS) and the rate of major postoperative complications (Clavien-Dindo ≥3) were extracted from prospectively maintained databases.

**Results:**

Low SMI was present in 78.3 % and low MAI in 48.8 % of the patients. The multivariate analysis showed lymph node metastasis [hazard ratio (HR) 1.8; 95 % confidence interval (CI) 1.1–2.9], microscopic radicality (HR 2.0; 95 % CI 1.2–3.4), and low MAI (HR 2.0; 95 % CI 1.2–3.3), but not low SMI to be significantly associated with decreased OS. Low MAI (HR 1.9; 95 % CI 1.0–3.8) was the only independent risk factor for major postoperative complications.

**Conclusion:**

Skeletal muscle quality, but not muscle mass, predicted survival and major complications after PD for periampullary, nonpancreatic cancer. Preoperative CT-derived body composition measures may stratify patients into risk categories and support shared decision making.

**Electronic supplementary material:**

The online version of this article (doi:10.1245/s10434-016-5495-6) contains supplementary material, which is available to authorized users.

Recently, the value of computed tomography (CT)-derived body composition measures for predicting postoperative outcomes has gained interest. For example, loss of skeletal muscle mass shown on preoperative CT imaging has been associated with worse short- and long-term outcomes after resection of tumors of varying origin.^1^ Also in gastrointestinal and hepato-pancreato-biliary (HPB) surgery,^2^ including surgery for pancreatic cancer,^3^–^6^ low skeletal muscle mass has been associated with increased morbidity and mortality and worse survival. Less consideration has been given to muscle attenuation shown on CT imaging, which has been associated with worse survival for patients with solid tumors of respiratory and gastrointestinal origin, for patients with melanoma or metastatic renal cell carcinoma,^7^–^9^ and recently for patients with pancreatic cancer.^4^,^5^,^10^

Low muscle attenuation reflects decreased muscle quality by an accumulation of intramuscular lipid depositions (myosteatosis), and the presence of myosteatosis on preoperative CT imaging may be shown through a negative correlation with the amount of intramuscular adipose tissue.^11^–^13^ Both myosteatosis and loss of muscle mass lead to a decrease in muscle strength.^14^

Studies associating preoperative CT-derived body composition measures of patients having periampullary, nonpancreatic cancer with survival and major postoperative complications do not exist. However, periampullary cancer constitutes about one third of all patients undergoing pancreatoduodenectomy (PD).^15^ The 5-year survival rate after resection of periampullary (distal bile duct, papilla, and duodenum) cancer may reach 50 %, twice the rate for pancreatic cancer patients.^15^,^16^ Mortality rates after PD are about 1 to 3 % in expert centers, but postoperative morbidity occurs in up to 50 % of patients.^15^,^17^ Therefore, risk stratification and response prediction before treatment remain of great interest. We assessed the association of skeletal muscle mass and muscle attenuation (quality) with OS and major postoperative complications in patients undergoing PD for periampullary, nonpancreatic cancer.

## Patients and Methods

### Study Cohort and Data Acquisition

All patients who underwent PD between 2000 and 2012 for primary papilla of Vater (C24.1), extrahepatic bile duct (C24.0), or duodenal (C17.0) adenocarcinoma were selected from a prospectively maintained database at a single center. Patients with pancreatic ductal adenocarcinoma (C25) were excluded. Topography and morphology were coded according to the international Classification of Diseases for Oncology (ICD-O). The primary outcome was OS, defined as the time between PD and death. Patients were observed until death or 1 September 2015, at which time they were censored. Survival data was obtained from the Municipal Personal Records Database, the central registry for all Dutch inhabitants.

The secondary outcome was the rate of major postoperative complications, defined as any complication classified as Clavien-Dindo grade 3 or higher within 30 days after PD or during admission, whichever was longer.^18^ Overall morbidity was evaluated and consisted of surgical and nonsurgical complications. The incidence of major postoperative pancreatic fistula, postpancreatectomy hemorrhage, and delayed gastric emptying also were noted, all according to the International Study Group of Pancreatic Surgery definitions.^17^,^19^,^20^

### CT Image Analysis

The final preoperative CT scan was used to determine skeletal muscle mass, muscle attenuation, and adipose tissue area. Patients were excluded from the analysis if no preoperative CT scan was available for analysis or if the cross-sectional area of interest was not in the field of view.

Sagittal images of CT scans were selected at the level of lumbar-3 (L3) from the same contrast series by an experienced radiologist (C.Y.N.). The third lumbar vertebra region contains the psoas, paraspinal (erector spinae, quadratus lumborum), and abdominal wall muscles (external and internal obliques, rectus abdominus, transversus abdominus).

**Fig. 1:**
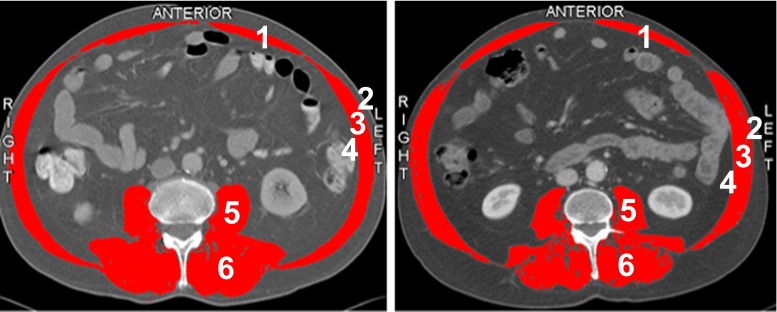
Computed tomography scans at the third lumbar vertebrae level of two male patients. *Right* Patient with a low skeletal muscle index (SMI, 56.8) (muscle mass) but a normal muscle attenuation index (MAI, 49.8) (muscle quality). *Right* Patient with a normal SMI and a low MAI (MAI, 24.0). The skeletal muscle area is highlighted in *red*. *1* rectus abdominis, *2* external oblique, *3* internal oblique, *4* transverse abdominal, *5* psoas, *6* paraspinal

The images were analyzed by a trained single observer (N.C.H.) using SliceOmatic V5.0 software (Tomovision, Montreal, QC, Canada). This software enables specific tissue demarcation using density thresholds by Hounsfield units (HU; Fig. 1). The observer was blinded to the patient’s postoperative course and survival.

To determine skeletal muscle mass at the L3 level, the cross-sectional skeletal muscle surface (cm^2^) was identified and quantified by HU thresholds of –29 to +150.^1^,^3^,^7^ Muscle area was normalized for height in meters squared (m^2^) and reported as lumbar skeletal muscle index (SMI) (cm^2^/m^2^). The muscle attenuation index (MAI) was determined by calculating the average HU at the L3 level. Intramuscular adipose tissue was additionally identified and quantified by HU thresholds of –190 to –30.^7^,^13^

### Statistical Analysis

To determine cutoff values for low SMI and MAI, we calculated sex-specific cutoff values by optimum stratification, as previously described.^1^,^21^,^22^ Optimum stratification is a statistical method that determines the threshold value of a continuous variable (SMI, MAI), which is based on log-rank statistics and best separates patients in terms of time to an event outcome (death).

Differences between groups were analysed using Pearson’s chi-square test and the independent *t* test as appropriate. Correlations between continuous variables were assessed using Pearson’s correlation coefficients. All *p* values were considered significant at the 0.05 level. Survival data are reported as median (range). OS rates, defined as the months of survival after PD, were calculated and compared using the Kaplan-Meier method. Conventional covariates included in the univariable survival analysis next to the dichotomous variables SMI and MAI were sex, age, American Society of Anesthesiologists (ASA) grade, tumor category, lymph node metastasis, tumor size, tumor grade, and resection margin status.^16^,^23^^–^ 27 The conventional covariates included in the univariate analysis for major postoperative complications next to SMI and MAI were sex, age, ASA grade, body mass index (BMI), diabetes mellitus, and tumor location.^28^ Characteristics with a *p* value lower than 0.10 in the univariate analysis were entered into multivariable models. Statistical analyses were performed using IBM SPSS statistics version 21 (IBM, Armonk, NY, USA).

## RESULTS

### Study Cohort

The study identified 281 patients. Patients were excluded when a CT scan was inadequate to determine SMI or MAI (*n* = 97, 35 %) or when patient height could not be retrieved (*n* = 18, 6 %). Consequently, 166 patients were analyzed. The baseline characteristics between included and excluded patients did not differ (Supplementary Table 1). The baseline characteristics for the patients with low SMI and normal SMI, and for those with low MAI and normal SMA are shown in Table 1. Some significant differences are observed in the baseline characteristics. The median interval between the final preoperative CT scan and the date of surgery was 55 days [interquartile range (IQR), 39–74 days].

**Table 1:** Characteristics of 166 patients receiving pancreatoduodenectomy for periampullary (nonpancreatic) cancer stratified by muscle mass and muscle quality

Characteristics	*N* 166	Normal SMI			Low SMI			*p* value	Normal MAI			Low MAI			*p* value
		*N*	Mean ± SD	%	*N*	Mean ± SD	%		*N*	Mean ± SD	%	*N*	Mean ± SD	%	
		36		21.7	130		78.3		85		51.2	81		48.8	
Sex								0.18							0.13
Male	104	26		72.2	78		60.0		58		68.2	46		56.8	
Female	62	10		27.8	52		40.0		27		31.8	35		43.2	
Age (cont.)	166		61.7 ± 11.4			65.7 ± 10.7		0.05		59.9 ± 10.7			70.0 ± 8.6		<0.001
ASA score								0.16							0.01
I–II	134	32		88.9	102		78.5		75		88.2	59		72.8	
III–IV	32	4		11.1	28		21.5		10		11.8	22		27.2	
BMI (kg/m^2^) (cont.)			27.0 ± 4.5			24.4 ± 3.5		0.001		23.8 ± 3.2			26.2 ± 4.2		<0.001
Diabetes mellitus								0.82							0.04
No	141	31		86.1	110		84.6		77		90.6	64		79.0	
Yes	25	5		13.9	20		15.4		8		9.4	17		21.0	
Tumor location								0.36							0.7
Ampulla	83	17		47.2	66		50.8		44		51.8	39		48.1	5
Distal CBD	65	17		47.2	48		36.9		31		36.5	34		42.0	
Duodenum	18	2		5.6	16		12.3		10		11.8	8		9.9	
Tumor size (cont.)	150	30	2.6 ± 1.8		120	2.8 ± 1.9		0.58		2.7 ± 1.8			2.9 ± 1.9		0.49
Tumor grade								0.94							0.37
Well differentiated	8	2		5.6	6		4.6		6		7.1	2		2.5	
Moderately differentiated	90	20		55.6	70		53.8		46		54.1	44		54.3	
Poorly differentiated	68	14		38.9	54		41.5		33		38.8	35		43.2	
Tumor								0.61							0.62
Stage I	51	12		33.3	39		30		29		34.1	22		27.2	
Stage II	98	19		52.8	79		60.8		48		56.5	50		61.7	
Stage III	17	5		13.9	12		9.2		8		9.4	9		11.1	
Microscopic radicality								0.008							0.16
R0	117	19		52.8	98		75.4		64		75.3	53		65.4	
R1	49	17		47.2	32		24.6		21		24.7	28		34.6	

*SMI* skeletal muscle mass index, *MAI* muscle attenuation index, *SD* standard deviation, *cont.* continuous, *ASA* American Society of Anesthesiologists, *BMI* body mass index, *CBD* common bile duct, *R1* positive margin

The sex-specific cutoff values for low SMI were 53.5 cm^2^/m^2^ for males and 46.4 cm^2^/m^2^ for females. Low SMI was present in 130 patients (78 %). The sex-specific cutoff values for low MAI were 36.3 HU for males and 36.0 HU for females. Low MAI was present in 81 patients (49 %). Low MAI was negatively correlated with intramuscular adipose tissue area (*r* = –0.65; *p* < 0.001).

### Overall Survival

The median follow-up period for the patients who were alive on 1 September 2015 was 71 months. The median survival time for the patients receiving PD for periampullary carcinoma was 44 months (range 0–181 months). During the follow-up period, 91 patients (55 %) died.

**Fig. 2:**
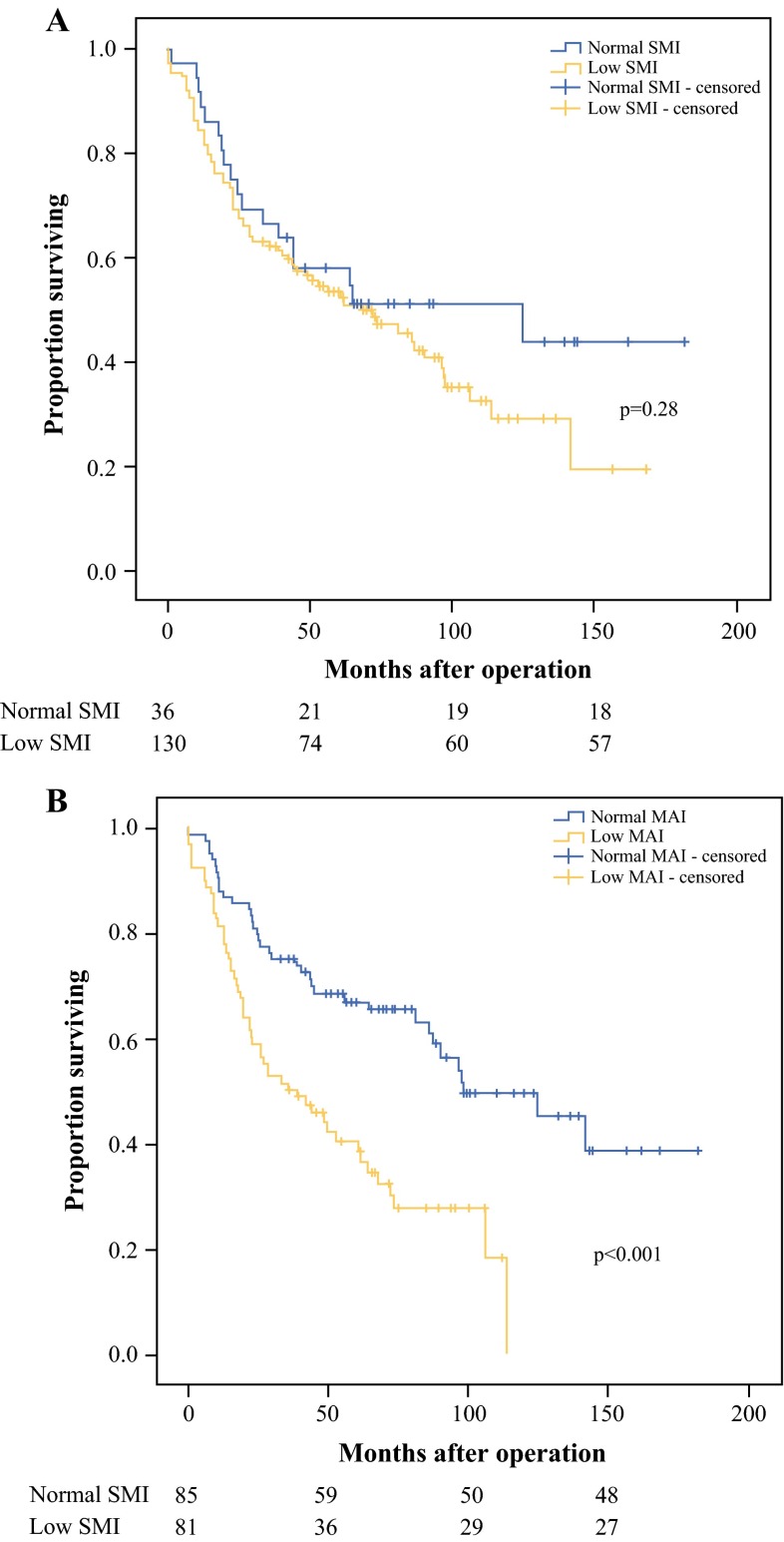
Overall survival rates after pancreatoduodenectomy for periampullary, nonpancreatic cancer according to **a** skeletal muscle index and **b** skeletal muscle attenuation

The OS curves for the patients with low SMI and normal SMI and those with low MAI and normal SMI are shown in Fig. 2. The median OS for the patients with low SMI was 72 months, which did not differ from the median OS of 124 months for the patients with normal SMI (*p* = 0.28; Fig. 2a). The median OS for the patients with low MAI was 39 months, significantly lower than the median OS of 98 months for the patients with normal MAI (*p* < 0.001; Fig. 2b).

Low SMI was not associated with an increased hazard ratio (HR) of death [HR 1.3; 95 % confidence interval (CI) 0.8–1.2]. Low MAI was associated with an increased hazard ratio of death (HR 2.4; 95 % CI 1.6–3.8). Other factors associated with OS in the univariate analysis included age (HR 1.0; 95 % CI 1.0–1.1), ASA score of 3 or 4 (HR 1.7; 95 % CI 1.0–2.7), T3 tumor stage (HR 2.3; 95 % CI 1.2–4.6), lymph node metastasis (HR 2.5; 95 % CI 1.6–3.8), and microscopic radicality (HR 2.8; 95 % CI 1.8–4.2).

In the multivariable analysis, low MAI remained independently associated with decreased OS (HR 1.95; 95 % CI 1.2–3.3). The independent predictors of OS also included lymph node metastasis (HR 1.8; 95 % CI 1.1–2.9) and microscopic radicality (HR 2.0; 95 % CI 1.2–3.4) (Table 2).

**Table 2:** Multivariable Cox regression analysis of overall survival after pancreatoduodenectomy for periampullary, nonpancreatic cancer

Characteristics	*N* 166	Univariable			Multivariable		
		HR	95 % CI	*p* value	HR	95 % CI	*p* value
Female sex	62	0.70	0.46–1.05	0.09	0.71	0.46–1.10	0.13
Age (cont.)	166	1.03	1.01–1.05	0.003	1.01	0.98–1.03	0.52
ASA score 3–4	32	1.65	1.01–2.71	0.05	1.22	0.73–2.05	0.45
Tumor category							
T1		Ref	–		Ref		
T2		1.22	0.58–2.58	0.60	0.85	0.39–1.83	0.68
T3		2.34	1.18–4.64	0.02	1.21	0.57–2.59	0.63
T4		2.12	0.88–5.13	0.10	0.89	0.34–2.32	0.80
Lymph node metastasis^a^		2.48	1.63–3.77	<0.001	1.82	1.14–2.91	0.01
Tumor size		1.03	0.92–1.15	0.58			
Tumor grade							
Well differentiated	8	Ref	–				
Moderately differentiated	90	1.57	0.49–5.09	0.45	1.29	0.37–4.47	0.69
Poorly differentiated	68	2.75	0.85–8.91	0.09	1.80	0.52–6.21	0.35
Microscopic irradicality (R1)	49	2.75	1.80–4.23	<0.001	2.01	1.20–3.36	0.01
Low SMI	130	1.33	0.79–1.24	0.28			
Low MAI	81	2.44	1.58–3.77	<0.001	1.95	1.16–3.29	0.01

*HR* hazard ratio, *CI* confidence interval, *cont.* continuous, *ASA* American Society of Aaesthesiologists, *R1* positive margin, *SMI* skeletal muscle mass index, *MAI* muscle attenuation index

^a^There were no patients with distant metastasis

### Major Postoperative Complications

The incidence of overall morbidity, major complications, and International Study Group of Pancreatic Surgery (ISGPS) complications is compared between patients with low and normal SMI and patients with low and normal MAI in Table 3. After PD, the overall morbidity rate was 66.3 % (110 patients). The overall morbidity rate was similar between the patients with low SMI (66.9 %) and those with normal SMI (63.9 %) (*p* = 0.73). The overall morbidity was significantly higher for the patients with low MAI (75.3 %) than for the patients with normal MAI (57.6 %) (*p* = 0.02).

**Table 3:** Complications after 166 pancreatoduodenectomies for periampullary, nonpancreatic cancer

	Normal SMI (*n* = 36)* n* (%)	Low SMI (*n* = 130)* n* (%)	*p* value	Normal MAI (*n* = 85)* n* (%)	Low MAI (*n* = 81)* n* (%)	*p* value
Overall morbidity	23 (63.9 %)	87 (66.9 %)	0.73	49 (57.6 %)	61 (75.3 %)	0.02
Major complication^a^	16 (44.4 %)	62 (47.7 %)	0.73	31 (36.5 %)	47 (58.0)	0.005
Postoperative fistula grades B & C	9 (25 %)	38 (29.2 %)	0.62	21 (24.7 %)	26 (32.1 %)	0.29
Postpancreatectomy hemorrhage grades B & C	2 (5.6 %)	9 (6.9 %)	0.77	2 (2.4 %)	9 (11.1 %)	0.02
Delayed gastric emptying grades B & C	14 (38.9 %)	49 (37.7 %)	0.97	25 (29.4 %)	38 (46.9 %)	0.03

*SMI* skeletal muscle mass index, *MAI* muscle attenuation index

^a^Clavien-Dindo grade 3 or higher complication

The incidence of major complications was 47 % (78 patients). The incidence of major complications was statistically equal between the patients with low SMI (47.7 %) and those with normal SMI (44.4 %) (*p* = 0.73). The incidence of major complications was significantly higher for the patients with low MAI (58 %) than for the patients with normal MAI (36.5 %) (*p* = 0.005). The patients with low SMI and those with normal SMI had similar rates for grades B and C postoperative pancreatic fistula (25.0 vs 29.2 %; *p* = 0.62), grades B and C postpancreatectomy hemorrhage (5.6 vs 6.9 %; *p* = 0.77), and grades B and C delayed gastric emptying (38.9 vs 37.7 %; *p* = 0.97). The patients with low MAI experienced more grade B or C postpancreatectomy hemorrhage (11.1 vs 2.4 %; *p* = 0.02) and more grade B or C delayed gastric emptying (46.9 vs 29.4 %; *p* = 0.03). The rate for grades B and C postoperative pancreatic fistula was similar between the patients with low and high MAI (32.1 vs 24.7 %; *p* = 0.29).

Low MAI was associated with an increased hazard ratio of major postoperative complications (odds ratio [OR] 2.4; 95 % CI 1.3–4.5) but low SMI was not (OR 1.1; 95 % CI 0.5–2.4) . The only other factor associated with major complications in the univariate analysis was BMI (OR 1.1; 95 % CI 1.0–1.2). After adjustment for potential confounding factors, only low MAI retained an independent association with the occurrence of major postoperative complications (OR 1.9; 95 % CI 1.0–3.8) (Table 4).

**Table 4:** Uni- and multivariate regression analyses of major postoperative morbidity after pancreatoduodenectomy for periampullary, nonpancreatic cancer

Characteristics	*N* 166	Univariable			Multivariable		
		OR	95 % CI	*p* value	OR	95 % CI	*p* value
Female sex	62	0.99	0.53–1.85	0.97			
Age (cont.)	166	1.02	0.99–1.05	0.12			
ASA score 3–4	32	0.85	0.39–1.85	0.68			
BMI (kg/m^2^) (cont.)	166	1.10	1.01–1.20	0.03	1.07	0.98–1.17	0.13
Diabetes mellitus	25	0.87	0.37–2.04	0.75			
Tumor location							
Ampulla	83	Ref	–				
Distal bile duct	65	0.91	0.47–1.74	0.77			
Duodenum	18	1.41	0.51–3.93	0.51			
Low SMI	130	1.14	0.54–2.39	0.73			
Low MAI	81	2.41	1.29–4.50	0.006	1.93	1.01–3.77	0.049

*OR* odds ratio, *CI* confidence interval, *cont.* continuous, *ASA* American Society of Anesthesiologists, *BMI* body mass index, *SMI* skeletal muscle mass index, *MAI* muscle attenuation index

## Discussion

In this first study investigating CT-derived body composition measures in patients undergoing PD for periampullary, nonpancreatic cancer, low muscle quality, but not low muscle mass, was independently associated with decreased survival and an increased incidence of major postoperative complications.

Various well-established patient and tumor characteristics are known to affect short- and long-term postoperative outcomes for patients with pancreatic and periampullary cancer.^16^,^23^–^26^ Our study adds to the previously identified risk factors for these patients, with a prognostic value equalling that of other conventional covariates.

The recent international interest in CT-derived body composition measures has led to identification of muscle mass and muscle quality as new independent predictors of outcome, with growing evidence to validate their prognostic impact.^2^ After some very recent studies investigating pancreatic cancer,^3^–^6^,^10^ our study is a first step toward determining the use of preoperative CT-derived body composition measures to stratify all patients receiving PD for cancer into risk categories and to assist in shared decision making.

Ideally, treatment of low muscle quality would lead to improved postoperative outcomes. Unfortunately, no such treatment is currently available.^1^ A randomized controlled trial with 52 nonsurgical, elderly patients demonstrated the clear effects of moderate physical exercise on age-related decline in muscle strength, but it involved a 12-month training program of moderate physical activity^29^ and therefore has no relevance in the preoperative setting or during neoadjuvant treatment. However, several prehabilitation strategies consisting of physical exercise have demonstrated improved postoperative outcomes.^30^ The effect of preoperative muscle or total-body exercises on postoperative outcomes for patients with low muscle mass or low muscle quality should be subject to future investigation.

Until recently, very little research on skeletal muscle attenuation (quality) was conducted. Some recent studies have investigated the prognostic significance of low muscle attenuation for patients with pancreatic cancer.^4^,^5^,^10^ One study included 230 patients undergoing PD, distal pancreatectomy, or total pancreatectomy for pancreatic ductal adenocarcinoma and identified muscle attenuation as an independent predictor of both worse survival and recurrence-free survival.^4^ In another study of 104 pancreatoduodenectomies, skeletal muscle attenuation was an independent predictor of severe complications according to the National Surgical Quality Improvement Program (NSQIP).^5^ It was not described whether periampullary cancer in the final pathology diagnosis was included in this study. Recently, attenuation of the psoas muscle on preoperative CT scan also was identified as an independent predictor of 1-year mortality for 518 elderly patients receiving HPB surgery, including pancreatic resection.^10^ In the limited number of studies available, skeletal muscle quality was an important covariate in the prognosis of patients receiving PD for cancer.

Previous studies have identified muscle mass as an independent predictor of postoperative complications and survival and also after resection of pancreatic cancer.^1^–^6^ In one of the earlier, large series after resection of pancreatic adenocarcinoma, muscle mass was not associated with the risk of overall morbidity or serious complications but was associated with an increased risk of 3-year mortality.^3^ To our surprise, we did not find an association between muscle mass and postoperative major complications or survival in the univariate analysis. It is unlikely that the influence of muscle mass on postoperative outcomes is different after resection of periampullary cancer compared with pancreatic cancer, although the incidence of complications does differ between the two groups.^28^,^31^,^32^ It is unclear whether the unequal distribution of patients between low muscle mass (78 %) and normal muscle mass (22 %) could be a reason for this finding.

We used the preferred and accepted method (optimal stratification) to find the most significant cutoff for L3 muscle index, as previously described.^1^,^21^ We found different cutoffs for men and women, which suggests that other, “static” cutoffs do not suit our cohort, so we believe that using these cutoffs would lead to inaccurate effect measurement. However, also when other previously used cutoff values for low muscle mass were applied, muscle mass still showed no effect on survival.^2^ The patients were better distributed among the muscle quality categories (51 vs 49 %). This suggests that some patients had a low muscle mass, but with normal muscle quality.

We found a significant negative correlation between MAI and intramuscular adipose tissue on preoperative CT scan, which reflects an accumulation of intramuscular lipid content.^11^–^13^ A higher muscle lipid content has been related to loss of muscle strength.^14^ This may explain why muscle quality and not muscle mass was an independent predictor of survival and major postoperative complications.

Complications after pancreatic surgery are very common.^17^,^19^,^20^ After resection for periampullary cancer, complications may occur even more frequently than after resection for pancreatic adenocarcinoma.^28^,^31^,^32^ The incidence of overall and major morbidity did not differ between the patients with low and normal muscle mass. The patients with low muscle quality experienced overall and major complications more frequently than the patients with normal muscle quality. These findings are reflected in our identification of low muscle quality as an independent predictor of survival and major complications.

We used several of the strongest and most frequent covariates predicting postoperative outcomes after pancreatic cancer surgery.^16^,^23^–^26^ Unfortunately, the presence of perineural or perivascular invasion was documented only incidentally by the pathologist and could therefore not be incorporated into our analysis.

We found a relatively long interval (median 55 days) between the final preoperative CT scan and the date of surgery. Possibly, as patients are referred to our tertiary hospital including CT scan, the interval is increased due to first discussion of the patient within a multidisciplinary team meeting, after which the operation is planned. Findings have shown that scans performed at a longer interval before surgery increases the incidence of intraoperative metastatic disease.^33^ Patient and event numbers were unfortunately too small to determine whether results would be different if patients who had a scan performed more than 30 days before the surgery were excluded.

This study had some limitations. We had to exclude a relatively high number of patients due to inadequacy of CT scans. However, the baseline characteristics did not differ significantly between the included and excluded patients (Supplementary Table 1). Furthermore, we identified some differences in baseline characteristics, mainly, a lower R1 rate for the patients with a low muscle mass and a higher BMI for the patients with low muscle attenuation than for the other patients. However, we corrected for these and other possible confounders in our analyses. Finally, we included three periampullary cancer types in our cohort. Survival and the incidence of complications may differ between the three types of periampullary tumors (duodenum, papilla, and distal bile duct tumors).^16^,^28^ However, in the sensitivity analysis, including also the cancer type in the multivariable analyses did not influence survival or the rate of major complications (data not shown).

## Conclusion

In a large cohort of patients undergoing PD for periampullary, nonpancreatic cancer, low muscle quality, but not low muscle mass, predicted poor survival and major postoperative complications. Preoperative CT scans contain valuable information on patient body composition that may improve preoperative risk assessment and can support shared decision making.

## Electronic supplementary material

Below is the link to the electronic supplementary material.

Supplementary material 1 (DOCX 15 kb)
