# Supplementary material for: Skeletal Muscle Quality is Associated with Worse Survival After Pancreatoduodenectomy for Periampullary, Nonpancreatic Cancer
Source: Ann Surg Oncol. 2016 Sep 8;24(1):272–80. doi: 10.1245/s10434-016-5495-6 (PMC5179584; doi:10.1245/s10434-016-5495-6)
Supplement: Supplementary file 1 — Supplementary material 1 (DOCX 15 kb) [file 10434_2016_5495_MOESM1_ESM.docx]

**Supplementary table.** Patient demographics and clinical characteristics, included vs excluded patients

|  |  | Included | | | Excluded | | |  |
| --- | --- | --- | --- | --- | --- | --- | --- | --- |
| Characteristics | N  281 | N  166 | Mean ± SD | % | N  115 | Mean ± SD | % | P value |
| Sex  Male  Female | 181  100 | 104  62 |  |  | 77  38 |  |  | 0.46 |
| Age (cont.) |  |  | 64.8 ± 11.0 |  |  | 63.9 ± 9.8 |  | 0.47 |
| ASA score  I - II  III - IV | 228  53 | 134  32 |  |  | 94  21 |  |  | 0.83 |
| BMI (kg/m²) (cont.) |  |  | 25.0 ± 3.9 |  |  | 25.5 ± 4.1 |  | 0.30 |
| Diabetes Mellitus  No  Yes | 238  42 | 140  25 |  |  | 98  17 |  |  | 0.93 |
| Tumor location  Ampulla  Distal CBD  Duodenum | 146  111  24 | 83  65  18 |  |  | 63  46  6 |  |  | 0.24 |
| Tumor size (cont.) |  |  | 2.8 ± 1.8 |  |  | 2.7 ± 1.4 |  | 0.84 |
| Tumor grade  Well differentiated  Moderately differentiated  Poorly differentiated | 15  156  110 | 8  90  68 |  |  | 7  66  42 |  |  | 0.72 |
| Tumor stage  Stage I  Stage II  Stage III  Stage IV | 82  176  22  1 | 51  98  17  0 |  |  | 31  78  5  1 |  |  | 0.13 |
| Microscopic radicality  R0  R1  R2 | 201  79  1 | 117  49  0 |  |  | 84  30  1 |  |  | 0.41 |

ASA, American Society of Anaesthesiologists; BMI, Body Mass Index; MAI, Muscle Attenuation Index; R1, positive margin; SMI, Skeletal Muscle Mass Index.
